# Persistent frequent attenders in primary care: costs, reasons for attendance, organisation of care and potential for cognitive behavioural therapeutic intervention

**DOI:** 10.1186/1471-2296-13-39

**Published:** 2012-07-06

**Authors:** Richard Morriss, Joe Kai, Christopher Atha, Anthony Avery, Sara Bayes, Matthew Franklin, Tracey George, Marilyn James, Samuel Malins, Ruth McDonald, Shireen Patel, Michelle Stubley, Min Yang

**Affiliations:** 1 Psychiatry and Community Mental Health, University of Nottingham, University Park, Nottingham, NG7 2RD, United Kingdom; 2 Primary Care, University of Nottingham, University Park, Nottingham, NG7 2RD, United Kingdom; 3 Research Cognitive Therapist, University of Nottingham, University Park, Nottingham, NG7 2RD, United Kingdom; 4 CLAHRC NDL, University of Nottingham, University Park, Nottingham, NG7 2RD, United Kingdom; 5 Division for Social Research in Medicines and Health, The School of Pharmacy, University of Nottingham, University Park, Nottingham, NG7 2RD, United Kingdom; 6 Economics of Health and Social Policy, University of Nottingham, University Park, Nottingham, NG7 2RD, United Kingdom; 7 Nottinghamshire Healthcare NHS Trust, 3 Oxford Street, Nottingham, United Kingdom; 8 Professor of Healthcare Innovation and Learning, University of Nottingham, University Park, Nottingham, NG7 2RD, United Kingdom; 9 University of Nottingham, University Park, Nottingham, NG7 2RD, United Kingdom; 10 Medical Statistics, University of Nottingham, University Park, Nottingham, NG7 2RD, United Kingdom

**Keywords:** High utilisers of care, Primary care, Cognitive behavior therapy, Hypochondriasis, Somatoform disorders, Health care economics and organizations

## Abstract

**Background:**

The top 3% of frequent attendance in primary care is associated with 15% of all appointments in primary care, a fivefold increase in hospital expenditure, and more mental disorder and functional somatic symptoms compared to normal attendance. Although often temporary if these rates of attendance last more than two years, they may become persistent (persistent frequent or regular attendance). However, there is no long-term study of the economic impact or clinical characteristics of regular attendance in primary care. Cognitive behaviour formulation and treatment (CBT) for regular attendance as a motivated behaviour may offer an understanding of the development, maintenance and treatment of regular attendance in the context of their health problems, cognitive processes and social context.

**Methods/design:**

A case control design will compare the clinical characteristics, patterns of health care use and economic costs over the last 10 years of 100 regular attenders (≥30 appointments with general practitioner [GP] over 2 years) with 100 normal attenders (6–22 appointments with GP over 2 years), from purposefully selected primary care practices with differing organisation of care and patient demographics. Qualitative interviews with regular attending patients and practice staff will explore patient barriers, drivers and experiences of consultation, and organisation of care by practices with its challenges. Cognitive behaviour formulation analysed thematically will explore the development, maintenance and therapeutic opportunities for management in regular attenders. The feasibility, acceptability and utility of CBT for regular attendance will be examined.

**Discussion:**

The health care costs, clinical needs, patient motivation for consultation and organisation of care for persistent frequent or regular attendance in primary care will be explored to develop training and policies for service providers. CBT for regular attendance will be piloted with a view to developing this approach as part of a multifaceted intervention.

## **Background**

In primary care, the top three per cent of face to face attenders with a general practitioner (GP) account for fifteen per cent of all consultations [1]. Frequent attenders (FA) generate five times as many prescriptions and hospital contacts compared to less frequent attenders [2]. Frequent attendance defined as the top 10 per cent of attenders in primary care is discriminative from normal attenders in terms of patient characteristics compared to broader definitions [3]. However, while only one in seven FA (top ten per cent of attenders age and gender adjusted) over one year continue to be FA over the next year, frequent attendance extending over two years usually then persists for a further year [4]. Frequent attendance is more common in some practices than others and varies from doctor to doctor, but most FAs consult all doctors in the practice so a practice based initiative is required to limit and quantify the effects of frequent attendance [5].

Frequent attenders consult practices with medically appropriate problems such as injuries at the same rate as other patients of the same age and gender but they consult more often for other problems such as functional somatic symptoms [6], and mental disorder such as depressive and anxiety episodes [7-15]. Psychological characteristics associated with FA include a history of childhood abuse or neglect and a history of childhood illness exposure (in self or members of family) [13]. Compared to other attenders, FAs have less sense of coherence [16], and greater health anxiety and hypochondriacal beliefs [17,18].

### **Reasons for frequent attendance**

Qualitative studies have thrown some light on the function that frequent consultation serves for the patient. One analysis from the UK suggests FAs have a set of idiosyncratic ground rules for consultation depending on their: perception of the GP’s role (e.g. frequent attendance was justified because the GP was employed to talk to, reassure, help and advise them); 2. relationship with the GP (e.g. less likely to consult so frequently if they thought the GP considered them to be a nuisance or hypochondriacal); 3. perception of their symptoms and past experience and knowledge of them; health anxiety where the fear of consulting outweighed the fear of not consulting; passivity and external locus of control – believing their consulting behaviour and ability to control some of their symptoms was beyond their control; mental health problems such as depression increased consultation behaviour; decision to consult being corroborated by family and friends or having desirable outcomes e.g. quicker return to work; and their familiarity with the processes for gaining access to the GP [19]. The frequency of consultation was much more likely to be increased if several of these factors were present together and less likely to occur if symptoms came under control, if they consulted elsewhere e.g. chiropractor, or if there were changes in life circumstances.

In another UK qualitative study [20], two patterns of consultation were discerned: a larger group who were unaware how frequently they had attended and a second group who related their frequent attendance to a particular crisis in their life that would pass. In general, patients who frequently attended viewed the GP as a respected authority figure who is the most appropriate figure to consult for distinctive and extensive physical symptoms that the patient perceived to require medical care and reassurance. They often described a relatively high number of physical sensations that were difficult for them to endure and required reassurance from their family doctor. Despite holding the doctor in high esteem, most frequently attending patients expressed some dissatisfaction with their treatment, typically in relation to the lack of understanding of their problems by the GP, the lack of an explanation for their problems that they could endorse and the progress made in achieving a resolution of their problems.

In a third qualitative study of frequent attenders with medically unexplained symptoms (MUS) from the United States [21], three groups of patients were identified: 1. patients who despite psychological insight about causes of MUS had neither reduced the intensity of their symptoms nor GP attendance; 2. patients with little psychological insight and capacity to help themselves who had vague symptoms and a strong sense of entitlement to be excluded from normal social roles; 3. patients with high health anxiety and concern that a diagnosis may have been missed who tended to be dissatisfied with their health care and focussed on their symptoms.

Although these quantitative and qualitative studies provide insights to the problems that frequently attending patients face and the conditions under which frequent attendance might occur, they have confounded patients whose frequent attendance is temporary rather than persistent, and they have not systematically linked their findings to opportunities for therapeutic intervention such as cognitive behaviour therapy. For instance the high prevalence of depression and anxiety disorders in these patients is not that informative given that most of these patients have already received and may still be taking antidepressant medication.

A common explanation for increased health care seeking behaviour is the development of health anxiety [22-26], sometimes arising from previous personal or family experience of significant health problems that were not initially diagnosed or managed optimally. Consequently, medical assertions that there is nothing seriously wrong are not reassuring [22]. Health anxiety increases muscle tension that might be experienced as pain and stress related cortisol leading to symptoms such as fatigue, and autonomic overactivity producing symptoms such as palpitations, breathlessness, faintness and diarrhoea. Unless doctors explain and normalise the presence of these symptoms, investment in their importance through investigations, referrals and prescriptions is likely to maintain or increase the health anxiety [27,28].

Another long-standing theory for frequent attendance is somatisation which refers to the presence of somatic symptoms that cannot be adequately explained by organic findings [28]. It tends to be used in relation to MUS, functional somatic symptoms or functional somatic syndromes such as chronic widespread bodily pain or the somatic presentation of underlying mental disorder such as depression presenting as back pain [28]. Somatisation tends to be associated with negative views about mental health, emotional and social issues as being issues of personal weakness and responsibility rather than an issue for health services. The function of somatisation may therefore be blame avoidance and is more common in people who come from families that hold similar views and personal experience involving violation of trust in authority e.g. abuse and not being believed or being helped with abuse [29-31]. For these reasons people with somatisation in any of its forms can be difficult to engage in treatment approaches that have an overt mental health focus leading to poor treatment take up [31,32], and when they are engaged in interventions, frequent attenders in primary care are usually underrepresented e.g. [33].Therefore studies are required that make a particular effort to recruit frequently attending patients in primary care.

### **Intervention studies**

Until recently there was conflicting evidence whether interventions for frequent attenders in primary care improved patient outcomes and reduced healthcare utilisation. Most studies defined frequent attendance over 12 months and in such patients clinical improvement and health care utilisation often improve with standard care over the next 12 months [3]. However, recent trials show evidence of improvement in mental health outcomes and cost effectiveness in patients who were defined as frequent attenders over two years with depressive episodes [34,35] or frequent attenders over two years with MUS [36]. These interventions employed an extensive stepped care intervention involving patient education, antidepressant treatment, monitoring of adherence to medication or nurse delivered antidepressant medication, reduction of analgesics, anxiety management and rehabilitation respectively.

The simplest effective intervention with frequent attenders with somatoform disorders has been the consultation letter [37]. Care within the practice is organised so one lead GP initiates a programme of care for the patient. After a careful clinical assessment involving history taking, physical and mental state examination and investigations, the GP explains that the patient has a problem with “somatisation” where the patient has an excessive focus on their body and becomes worried when there are changes in their body. The GP offers to see the patient on a frequent basis for a limited period to check that this is the case and to gain the patient’s trust. The GP tells the patient that no investigations will be carried out unless new symptoms develop and a physical examination indicates that further investigation is clinically warranted. After four to six weeks, appointments are decreased to monthly then two monthly with the same GP. The practice organises care so all requests for urgent appointments or to see other GPs are discussed with the lead GP and are strongly discouraged unless there is an urgent clinical indication. Randomised controlled trials (RCTs) of this intervention demonstrate reduced health care utilisation and increased physical function in patients who frequently attended with somatisation but have not improved mental health, social function outcomes or the perception that they remain unwell [38,39].

An intensive training course for GPs lasting 15 hours with regular supervision by mental health professionals reduced attendance over one year in a sample of 209 Spanish frequently attending patients [40]. The intervention formulated hypotheses for each patient’s frequent attendance based on organisation of care, doctor-patient relationship, social, cultural, mental or physical health problem grounds.

Although the benefits of a cognitive behaviour therapy intervention are now well established for health anxiety [25,41] and there is some evidence for its benefit in somatisation with frequent attendance [42], it has not been formally tested in relation to helping primary care doctors manage persistent frequent attendance. Despite the identification of reasons for frequent attendance by previous studies, the reasons are not tailored to the needs of the individual patient that the GP is treating. Cognitive behaviour therapy has the potential to formulate specific processes underlying why an individual frequently attends on a day to day basis, and to provide the GP and practice with a tailored plan to manage each persistent frequent attender [43].

### **Organisation of care**

In England, there has been a financially incentivised change in the organisation of more structured primary care for some physical disorders such as diabetes and cardiovascular disease identified in the Quality Outcomes Framework [44]. There is debate about how much these financial incentives have changed the process and outcomes of such care [45,46]. In contrast the same organisation of care does not routinely happen in the management of mental disorders although there are additional psychological treatment resources to help primary care manage depression and anxiety [47]. Furthermore, the interventions that have demonstrated clinical and economic benefits in persistent frequent attenders with depression or functional somatic symptoms required considerable reorganisation of care and workforce training and development. Yet most practice staff, other than GPs, have no formal training in the assessment and management of mental disorders, and few GPs have training in the management of functional somatic symptoms or persistent frequent attendance [48]. There is a need for specialist mental health support for practice staff managing these patients [49] but the current tariff system does not reward mental health staff to do such work unless there is face to face contact with the patient [50].

Despite the evidence for high consumption of secondary health care resources among persistent frequent attenders in primary care, there has been no formal economic study of persistent frequent attendance. Without such a study, the potential costs and benefits of mental health intervention to support primary care doctors to manage these patients cannot be properly planned and subsequently evaluated. Furthermore there is a need to understand how different practices organise the care for these patients and how patients make their decisions on who to consult in order to tailor interventions for persistent frequent attenders could be applied to a wide range of practices in England.

### **Terminology**

The research literature uses the term “frequent attender” which has a variety of definitions from study to study. We have used this term in the above review. However, a panel of service users reviewing our study documents found the term “frequent attender” to be offensive as it implies a criticism of the patient for attending too often. The term may be seen as pejorative and blaming the patient [19-21,51]. Instead the service user panel recommended a more neutral term “regular attender” which we now use below.

### **Study objectives and hypothesis**

#### **Objectives**

The primary objective is to gain a better understanding of the needs and implications for the health service of regular attenders in primary care in terms of the costs of health care, patterns and chronicity of health care consultation, clinical characteristics, reasons for consultation, and organisation of their care by the GP and practice.

Secondary objective are to:

1. Estimate the cost to the health service of regular attendance in primary care and to examine the pattern and chronicity of regular attendance.

2. Clinically characterise people with regular attendance in primary care compared to normal attendance.

3. Explore motivations, reasons for regular attendance in primary care and patient experiences of consultation.

4. Explore practice characteristics, organisation of care and other barriers and drivers on the management of regular attenders and other complex patients with multiple morbidity.

5. Use CBT formulation to understand the development of regular attendance and its relationship to other health and social problems;

6. Develop a descriptive typology of the development and reasons for regular attendance using information derived from the qualitative interviews and CBT formulation;

7. Explore the acceptability, feasibility and clinical utility of CBT formulation and a treatment plan delivered jointly by the practice and the cognitive behaviour therapist for regularly attending patients.

#### **Hypotheses**

1. Most patients with regular attendance in primary care show chronic patterns of health care use with a duration of three or more years.

2. Compared to age, gender and practice matched normal attender controls, regular attenders are more likely to have depressive and anxiety disorders, somatoform disorders, high health anxiety, two or more active physical health problems, and a history of trauma or abuse in childhood or adulthood.

3. Compared to normal attender controls, regular attenders will incur up to five times the health care costs over the preceding 10 years but have a lower quality of life on the EQ5D.

4. Cognitive behaviour formulation and joint treatment between a cognitive behaviour therapist and a GP are feasible, acceptable and have clinical utility for regular attendance.

#### **Method**

The study was given full ethics approval by the Nottingham 1 NRES Committee (reference number 11/EM/0392).

#### **Study design**

**Figure 1:**
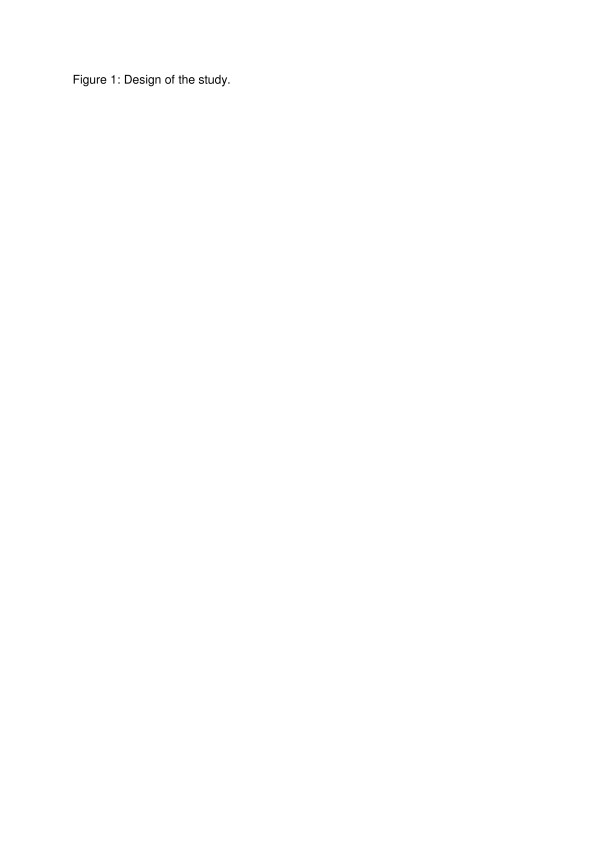
Design of the study.

The study consists of four parts (Figure 1):

1. A clinical and economic case control study of regular attenders and normal attenders including examination of health care records over the previous 10 years.

2. A qualitative study of patient’s reasons for regular attendance.

3. A qualitative study of primary care practice staff to explore the organisation of care for regular attenders.

4. A feasibility, acceptability and clinical utility study of cognitive behaviour therapy formulation and treatment alongside joint patient management between the GP and therapist in regular attenders.

#### **Selection of practices**

Five practices will be purposefully selected to obtain diversity in existing organisation of care and the populations served by practices so that the assessment of need and pilot intervention may be generalised to other primary care practices in England. A large random sample of practices would not provide the opportunity to carry out detailed work in each practice. Practices will be sought to include a range reflecting differing registered patient list size, patients of differing social and ethnic diversity, and affluence or deprivation; and differing practice organisational contexts, including size of practice team, salaried GPs or GP partners; full and part-time staff configurations; approaches to patient access for appointments, continuity of care, and links with mental health services.

#### **Definition of regular attendance**

Face to face attendance with the GP on 30 or more occasions in two years, excluding routine health monitoring such as international normalised ratio (INR) monitoring for patients taking warfarin, defined a group of patients who were in the top ten per cent of attenders for each of the two years [4] in two differing pilot practices. Patients with catastrophic physical illness such as cancer and serious mental illness such as schizophrenia were not represented, indicating exclusion criteria in relation to catastrophic physical illness or serious mental illness were unnecessary.

### **Case control study of regular attenders and normal attenders**

#### **Inclusion criteria**

1. Regular attenders

· 30 or more face to face contacts with GP within the last 2 years established from the practice electronic record.

· Aged 18 years old and over.

· Consents to take part in the study

2. Normal attenders

· Between six and 22 face to face contacts with GPs over two years, established from the practice electronic record. A previous study of 828 consecutive attenders from eight Nottinghamshire practices revealed a median attendance of eight face to face consultations with GPs with approximately two thirds of the sample consulting between three and eleven consultations. Therefore we selected a normal consulting control group from patients who attended face to face appointments with the GP between six and 22 occasions.

· Aged over 18 years or older

· Consents to take part in the study

#### **Exclusion criteria for both regular and normal attenders**

· Contacts for routine health care checks e.g. INR monitoring, blood pressure monitoring, changes of dressings

#### **Recruitment**

Regular attenders will be identified from each practice and matched by age, gender and practice to a normal attender to control for practice and socio-demographic characteristics, by practice staff who will write to the patients to request their participation in the study. Approximately 100 regular attenders and 100 normal attenders will be recruited. Pilot work shows that 30-40% of regular attenders will agree to be interviewed but a higher proportion of normal attenders will agree to be interviewed. The representativeness of the selected regular attenders will be compared to the overall sample of regular attenders in the practices and the overall patient lists in terms of age and gender among those aged 18 years or over.

#### **Procedures and measures**

Practice staff will identify patients who meet the inclusion/exclusion criteria from their primary care practice electronic record. All eligible regular and normal attending patients will be invited to have a baseline interview (see below). Practice staff will send eligible patients a study pack containing an invitation letter (on practice letterhead) inviting them to take part in the study, an information sheet, describing the study, a ‘consent to contact’ form and a stamp addressed envelope. If patients are interested in finding out more about the study and speaking to a researcher, they will be asked to return the ‘consent to contact form’ to the study researcher in the stamped addressed envelope provided. The consent form will also ask the patient’s permission for the researcher to access their anonymised medical records even if they do not wish to be interviewed. A researcher will then telephone the patient to discuss the study further, answer any queries they may have, and if verbal consent is given, arrange a time for interview. One reminder letter (on practice letterhead) will be posted by practice staff to eligible participants who did not respond to the initial invite. In some instances where the GP feels the patient may be suitable for the study but the patient has not responded to invitations to take part, the GP may approach the patient directly and make an appointment to see them to explain the study.

Each patient will be interviewed to explore the presence or absence of psychiatric disorder including depressive disorder, anxiety disorder and somatoform disorder [52] using a structured psychiatric interview (SCID-1) [53], the presence and degree of severity of health anxiety over the last week on the Health Anxiety Inventory (HAI) [24], quality of life on the EQ5D [54], and information on health and social care, work and benefits using the Client Receipt Service Inventory (CSRI) [55] (Table 1).

**Table 1:** Baseline interview

SCID	SCID-1 for DSM-IV (Structured Clinical Interview for Diagnostic and Statistical Manual of Mental Disorders)(First et al., 1997)
CSRI	CSRI Client Services Receipt Inventory (Beecham and Knapp 2001)
EQ5D	EQ5D Standardised instrument for use as a health measurement (Euroqol group 1990)
HAI Short Week	Health Anxiety Inventory (HAI, a 14 item self-rated measure of health anxiety, Salkovskis et.al, 2002) with a score above 21 indicating severe health anxiety.

In addition, information on active physical health problems currently and in the last two years, body mass index, smoking and alcohol consumption, and exercise will be extracted from practice records. The health care records will be examined 12 months later to ascertain health care use in the 12 month period following baseline assessment. Secondary health care use will be ascertained by exploring health care records over the preceding 10 years and prospectively over the next 12 months in all local secondary care services and nationally from Hospital Episode Statistics collected by the Department of Health for England.

### **Statistical analysis in case control study**

Burton et al. [56] showed that 93 (48%) of patients with MUS diagnosed by secondary care physicians on two or more occasions and referred at least three times in the last five years had depressive and anxiety disorder compared to 38 (25%) patients only referred once to secondary care in the previous five years (odds ratio 2.6 (95% confidence intervals 1.6, 4.1). If these groups are assumed to be similar to the regular attenders and normal attenders in the current study, a sample size of 100 regular attenders and 100 normal attenders will have 90% power to show a difference at the 5 per cent level of significance on two tailed testing.

The regular attender and normal attender groups will be compared on the proportion with mental disorder (depressive disorder, anxiety disorder, somatoform disorder, overall prevalence of mental disorder), mean number of active physical health problems, distribution of severity of health anxiety, proportion of obesity on BMI, mean scores on smoking, alcohol consumption and exercise, and utility related quality of life on the EQ5D. Univariate analysis to test differences between the two groups will be performed using *t*-test for group means or geometric means if logarithm transformation is required for variables with skewed distribution. Chi-squared test will be used for group differences in categorical variables. The accuracy of recall by patients over the previous three months will be compared to health care contacts recorded in the primary care record. There is some evidence that regular attenders may not accurately recall health care contacts and do not realise how frequently they are attending compared to practice records [21,51].

Multivariate regression analyses conditional on case–control matched pairs will be conducted to identify factors significantly associated with frequency of primary care contacts by regular and normal attenders separately. Important variables (either statistically significant or clinically important) for each group of patients will be used in a discriminant analysis which could further select a list of variables that best reflect different characteristics of the two groups and separate them in principle of a maximum distance. In regression analysis we will use generalised linear model (GLM) so that different dependent variables will be analysed in different type of model, i.e. linear regression for continuous variables, logistic for binary and multinomial for categorical variables.

### **Health economics in case–control study**

The study provides a unique opportunity to examine the health care costs longitudinally of this group of patients compared to a normal distribution of patients. The health and resource patterns for regular attenders have not been compared to the resource cost for a normal population before. This work will give a real opportunity to establish the true cost to society of a regularly attending group of patients. Whilst health economics does not generally consider burden of illness studies, unless the true resource costs of this group of patients are known, we cannot establish the likely willingness to pay for an intervention that may reduce the costs incurred by these regularly attending patients. Logically any intervention that costs less than the current annual treatment cost for regular attenders will at least probabilistically be potentially cost effective.

We will examine health and social care costs over the preceding 10 years from primary care records in both the regular attenders and normal attenders who have consented to the study. For each preceding year over the last 10 years, information will be collected on the number of contacts and missed appointments with the GP (at surgery, home or telephone contacts), practice nurse, community nurses, counsellors, physiotherapists, occupational therapists, social workers, community workers, NHS Direct, drop-in centres and home help, prescriptions, investigations, secondary care in-patient and out-patient care, and emergency care. Practice based primary care information is likely to be more accurately recorded than contacts that happened outside the practice. However we intend to trawl for the GP referral letters to obtain estimates of secondary care usage and we will also collect contact information for each participant from all local hospitals. Full and complete anonymised data sets of ten year medical records will be processed using a replicable method of electronic data collection that has been developed specifically to answer a number of health and economic and clinically important questions. In addition this data will provide us with a detailed insight into patients resource use in primary as well as secondary care. We will be able to check the data retrieved electronically with the data collected from the modified Client Services Receipt Inventory (CSRI) [55] and therefore check the degree to which care outside the practice may be underestimated. Similarly the analysis of reporting of contacts with primary care at interview compared to the primary care record will explore whether or not regular attenders are underreporting health contacts compared to normal attenders as reported previously [21,51]. To examine agreement in some key information of service use between patient recalls and primary care records, we shall use non parametric correlation analysis which includes Spearman’s rank correlation coefficient, Kendall tau, and Kappa coefficient for different outcome measures where it is appreciated. We will explore the effect any under-reporting by regular attenders may have had on health and social care costs.

We will ascertain health, social and personal costs using the modified CSRI [53] and also quality of life and cost utility on the EQ5D [54] over the three months preceding baseline interview in both the regular attender and normal attender control groups. Services measured will include secondary care mental and physical care, primary care, accident and emergency and other out of hours or direct access services such as NHS Direct, NHS walk-in centres, services provided by councils, other social care agencies, voluntary agencies and complementary therapy. In addition, inputs provided from family members as a result of the patient’s health problems will be recorded and the amount of time off work by the patient and any carer due to ill health will be recorded and costs attached. Other costs include out-of-pocket expenses (such as travel to seek health care), expenditure on 'over-the-counter' medicines, the extent of informal care (for children or the house) and any other costs (such as informal care, heating from being at home when off work and costs to carers). Data on social security payments will also be collected. Prescribing data will be obtained from primary care patient records and patient interview. Nationally applicable unit costs [57] will then be combined with the service use data to generate service costs for primary care. Medication costs will be obtained from the British National Formulary, and hospital based costs will be obtained from NHS reference costs/PBR (payment by results). Costs of time off work will be calculated from the patient’s own account of their salary and normal expectations of overtime and informal care to the patient will be calculated using the carer’s wage rate where possible or alternatively at the commercial rate that a carer would have normally expected to be earning at that time. Cost data will be combined with utility weighted health-related quality of life data as recorded by the EQ5D [54].

### Qualitative study of patients’ reasons for persistent regular attendance

A central hypothesis of the study is that most regular attendance is initiated by the patient in response to a perceived health problem and that health care seeking is a motivated behaviour that serves a function of high psychological importance to that individual and is also shaped by the experience of health care seeking. Our pilot study CBT case series of eight regular attenders confirms that uncertain and unclear communication by health professionals, and consultations with health professionals that do not address the regular attender’s main health concerns, are likely to increase health anxiety and result in further health care seeking. However, the representativeness of the participants who took part in the CBT case series suggests the need for a specific qualitative study involving maximum variance sampling of patient characteristics.

#### **Recruitment**

A sample of approximately 12 to 15 regular attending patients will be selected for individual qualitative interviews based on age, gender, ethnicity, practice, employment status, marital status, consultations mainly in primary care, emergency care and secondary care, high and low health anxiety, presence and absence of somatoform disorder, presence or absence of depressive disorder, presence or absence of panic disorder, presence or absence of more than one active physical health problem. Sampling will allow participants to meet more than one of these selection criteria. The exact number of participants will depend on the representation in this sampling frame and saturation of themes as they emerge. This purposive sample will be selected from the 100 regularly attending patients that have originally completed a consent to contact form and been recruited to the study. Selected participants will be invited to take part in the qualitative interview by the study researcher who will send them an invitation letter, information sheet and reply slip, along with a stamped addresses envelope. Upon receipt of the reply slip, the researcher will contact the participant, answer any questions they may have, and if verbal consent is given, a time and place for the interview to take place will be arranged. This might be at the participant’s home or at their general practice. Separate written consent for this part of the study will be sought.

### **Procedure and analysis**

The interviews will be carried out by an experienced qualitative interviewer in a private and quiet venue such as the patient’s home or GP surgery. The interviews will be semi-structured according to a topic guide, audio recorded, transcribed and analysed thematically using constant comparison. Catalytic validity will be achieved by using a reference group of researchers from a range of different backgrounds (academic GP, sociology, business school, psychiatry) so that the emerging data can be critically examined and the topic guide is refined according to the emerging thematic analysis. Recruitment of participants to interview will halt when saturation of themes has been achieved.

An individual’s behaviour can be interpreted in terms of its social function for that individual [26,48,50,58].Thus health care seeking can be seen as a motivated behaviour that serves a function of high psychological importance to that individual and is shaped also by the experience of health care seeking. Therefore there is value in understanding temporally the process of the evolution of the symptoms that led to a consultation and what processes are involved in the decision to seek a consultation. The following questions will be explored: How do these differ when a person seeks a consultation to when they do not seek a consultation for a particular set of symptoms? If they did seek a consultation, what were they seeking from the consultation and were those wishes met? How do consultations that meet their needs differ from those that do not? How does the decision to consult primary care and responses from primary care differ if it at all from the decision to consult secondary care or alternative providers?

### **Qualitative study to explore the organisation of care**

The qualitative study of primary care staff will explore how care is currently organised for regular attenders within primary care practices in contrast to other groups of patients such as those with chronic physical health problems or chronic mental disorders. It will explore barriers and drivers to the organisation of care across the practice as outlined in the stepped care interventions that have shown some evidence of effectiveness in randomised controlled trials. Such barriers might be at practitioner level (e.g. attitudes, skills, knowledge, incentives), practice level or policy level.

### **Procedure and analysis**

Two forms of data will be collected. In-depth interviews with purposively sampled participants including GPs, reception staff, practice nurses, practice managers and other primary care professionals will be audiotaped and transcribed. All practices will be sampled so inter-site comparisons can be made. A second body of data will be formed from practice policies, clinical guidelines, memos and any other documents relevant to this clinical group at a national, local or practice level.

Interview data will be analysed through a constant comparison approach. Following first level coding, newly obtained and previously obtained data will be constantly compared back and forth to form categories that will eventually form a narrative concerning the organisation of care of these patients. The information obtained from staff will be contrasted with accounts from patients of seeking help so that areas of agreement and disagreement between patients and different staff members can be explored as they arise in the analysis. Recruitment and analysis will continue until there is a saturation of themes. Data from organisational documents will be subject to content analysis and will be used to illuminate and contextualise the interview data.

### **Pilot intervention study**

The aims of the pilot intervention in the five practices are three fold to:

1. gain a better understanding of the development of regular attendance using CBT formulation (secondary objective 1);

2. develop a descriptive typology of the reasons for regular attendance and the opportunities for therapeutic intervention based on CBT formulation (secondary objective 2);

3. determine the acceptability, feasibility and clinical utility of joint care between the cognitive behaviour therapist and GP based on both the CBT formulation and treatment and a collaboratively agreed treatment plan (GP, cognitive behaviour therapist and patient) (secondary objective 3).

### **Intervention**

The intervention will be offered at one of two levels to all regular attender patients who complete the baseline assessment and case record examination outlined in case study one (Figure 1). It will not be offered to normal attenders. Level 1 intervention will be offered to all regular attenders provided they consent to further assessment and therapeutic intervention by one of three cognitive behaviour therapists, currently have functional somatic symptoms, depressive or anxiety disorders, do not have an urgent need for other health care and are not already receiving mental health care as shown in Figure 1.

**Figure 2:**
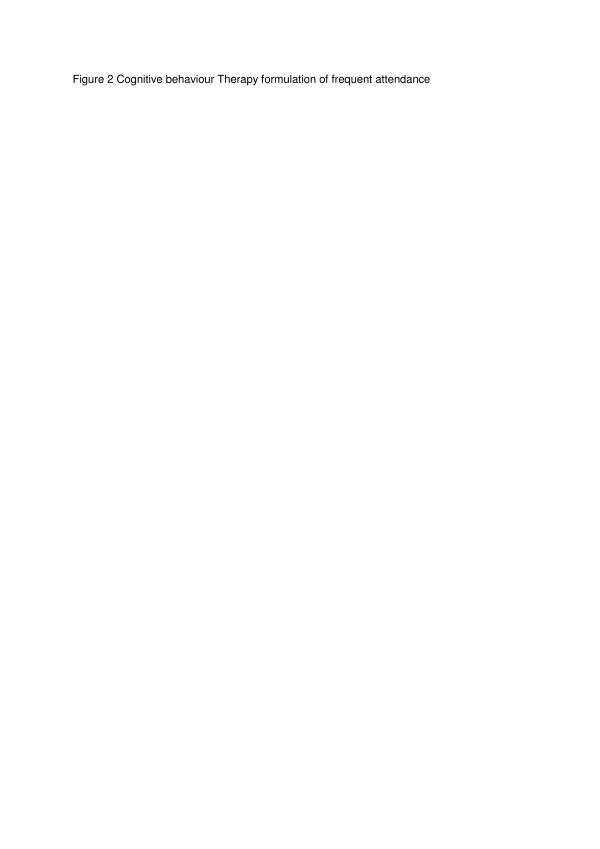
Cognitive behaviour Therapy formulation of frequent attendance.

In level 1 interventions the cognitive behaviour therapist will formulate the reasons for regular attendance in both primary care and secondary care settings in consultation with the GP who usually sees the patient (Figure 2). In keeping with recent developments in CBT formulation, the latest problem-specific models will be used to formulate or a generic model applied for cross-diagnostic issues [59]. Whichever CBT model is used emergent formulations and intervention plans will focus on addressing three areas: 1. The primary focus of the formulation is on the pattern of regular consultation behaviour of the patient with health services rather than somatic distress or health anxiety; 2. The formulation takes a developmental perspective seeking to understand how the regular consultation behaviour arose and is maintained [60]; 3. Consulting behaviour of the patient is understood primarily in terms of its social function from the patient’s perspective the responses of primary care, secondary care and social environment to the consulting behaviour.

An initial formulation may be developed in one or two sessions. However, in some cases a comprehensive formulation may take longer where there are issues of great interpersonal sensitivity. The cognitive behaviour therapist will then plan further management of the patient with the lead GP based on the formulation using established cognitive behaviour therapy strategies for managing functional somatic symptoms, health anxiety, depression or anxiety disorders. The management plan may involve therapeutic interventions organised by the GP such as antidepressant prescribing, a reduction of symptomatic prescribing e.g. opiate painkillers, hypnotic drugs to induce sleep, organisation of care within the practice so that the same practice staff see the patient [38], or the organisation of help with occupational, financial or social care problems [40]. The overall aim is to reduce the need for consultation, promote coping and self-efficacy as well as management of underlying health problems. The cognitive behaviour therapists will be supervised by RM and will also have access to supervision from an academic GP.

Level 2 interventions will be offered to all other regular attenders who refuse consent to treatment or are excluded from level one intervention because they are already receiving psychological treatment e.g. as part of a pain clinic or mental health care or they require other types of urgent care. The level 2 intervention will consist of a preliminary formulation of the reasons for regular attendance and a suggested management plan on the basis of the baseline assessment and examination of the practice record. Consent will be requested to feedback clinical information back to the GP. If the patient does not consent to such feedback then the case formulation will not be fed back to the GP unless the patient’s safety could be compromised e.g. high suicide risk. The detailed information obtained in the baseline assessment and records of the patients will often permit a CBT formulation although inevitably there will be more areas of uncertainty in the assessment and management plan for the GP to consider than if the patient agrees to further assessment and management by the cognitive behaviour therapist.

### **Measures**

Since this is a feasibility study of a complex intervention with a range of clinically important outcomes, a number of outcome measures will be collected over a six month period [61] to inform the design of future intervention studies. The small sample, non-randomised design of the study is unsuitable for making any definitive assessments of the clinical or cost effectiveness of the intervention so a primary outcome variable is not specified.

Outcomes measured at baseline and six months using postal questionnaires and telephone interview with all patients who consent to take part in the level one intervention:

1. The proportion of regular attenders who agree to take up a level one intervention and their characteristics compared to those who do not agree.

2. Attendance rates at appointments with cognitive behaviour therapists.

3. Patient satisfaction on a five item measure (met needs, help with problems, amount of time, overall satisfaction, want same treatment again) [62].

4. Change in the mental health and physical health component summary score of the 36-item self-rated SF-36 version 2 measure [63].

5. Cost utility or cost effectiveness over six months using the EQ5D [54] and costs primarily from both health and social care perspective.

6. Proportion who achieve a 4 point change in the mental health and physical health component summary scores (regarded as clinically significant change, 39) of the 36-item self-rated SF-36 version 2 measure [63].

7. Change in depression on the nine item PHQ-9 [64].

8. Change in anxiety on the seven item GAD-7 [65].

9. Change in health anxiety on the 18 item Health Anxiety Inventory [24].

10. Change in somatic symptoms as assessed by the PHQ-15 [66].

11. Patient-doctor relationship [67].

12. Two measures of cognitive process, the rumination scale [68] and the tendency to avoid thinking about painful emotional experiences [69].

### **Analysis secondary objective 1**

The cognitive behaviour therapy formulations derived by the cognitive behaviour therapist will be analysed thematically in relation to the development of regular attendance. Regular attendance may be related to distal factors e.g., childhood abandonment and neglect or to more proximal events e.g. life events or period of depression precipitating a change to regular attendance in primary and secondary care settings. The analysis will explore the interplay between the distal and proximal factors associated with regular attendance.

### **Analysis secondary objective 2**

The patterns of behaviour shown in relation to primary care and secondary care use will be explored in terms of amount of contact, which contacts are made, attendance at prearranged or same day appointments, and the reasons for attendance. The typology will be made thematically. The maintaining factors for the pattern of regular attendance and both short-term and medium term goals for treatment will also be assessed thematically.

### **Analysis secondary objective 3**

The primary analyses in relation to the acceptability and feasibility of the intervention is the uptake of the level 1 and level 2 interventions by regular attender patients, attendance at further level 1 intervention appointments with the cognitive behaviour therapist, and changes in patient satisfaction over 6 months by regular attender patients who consent to take part in the level one intervention. Descriptive statistics will be used to analyse uptake and attendance at the intervention. Changes in clinical outcomes will provide some evidence of clinical utility and to plan further intervention studies rather than to provide evidence of clinical or cost effectiveness. Analysis of change in satisfaction and clinical outcome from baseline to six months will be by intention to treat using two-sided significance tests with a random practice effect. We will examine time point and time*treatment interaction effects. Multiple imputation will be used to handle missing data with a sensitivity analysis employed for not missing at random.

As part of the qualitative interviews with individual regular attender patients and staff outlined above, the subjective experience of the acceptability and usefulness of the intervention by the cognitive behaviour therapist will be explored. Barriers and drivers to the delivery of the intervention will be explored to refine the intervention. Analysis will be conducted thematically as outlined previously.

## **Discussion**

In the current global financial climate where most governments are struggling to fund health services, there is a growing interest in the cost, clinical characteristics and interventions for high utilisers of care such as persistent frequent attenders in primary care. Previous studies indicate such patients are difficult to manage and will require changes in usual professional practice and organisation of care that may be resisted by patients and professionals alike [32,36,37,42,70]. Furthermore, there is scepticism among primary care professionals about the effectiveness of interventions for frequent attenders in primary care [48].

The current study addresses several important preliminary steps in developing a complex intervention that might be clinically and cost effective [61]. It explores the need for the intervention at an economic level and a clinical level. There is no specific economic study of the health care costs of persistent frequent attendance in primary care but it is necessary to consider this to establish the economic parameters for developing a therapeutic intervention. A study examining high utilization of care overall in a practice is likely to recruit mostly women of all ages and older men [71] so the results may not generalise to younger men who attend more frequently than their peers. Use of computerised scanning of data directly into economic databases from the large medical records held on these patients in primary and secondary care over a ten year period ensures that such an economic analysis is now feasible.

Previous studies have been descriptive (e.g. 6–15) but they do not offer enough specific information in relation to how mental health and other problems are related to the pattern of frequent consulting to develop effective interventions [21]. Cognitive behaviour therapy may provide a useful framework for understanding consulting behaviour as a motivated behaviour [22,59] that serves a function such as relieving distress for the frequently attending patient. It may also provide a framework for understanding how extreme consulting behaviour might arise from a developmental perspective and why it is maintained in the context of the person’s life and the responses of primary and secondary care. Understanding of how otherwise inexplicably high rates of consultation have arisen using the narrative context of the patient’s life story [72] may help the clinician to increase their empathy for the patient. Such empathy is necessary for strong enough therapeutic relationships to develop between the frequently attending patient, GP and cognitive behaviour therapist that might influence a patient’s behaviour without the need for coercion. The thorough analysis of factors maintaining consulting behaviour patterns may highlight opportunities for therapeutic intervention for the GP and cognitive behaviour therapist over both the short and medium term. Such an analysis may provide a theoretical basis for an intervention [61] if it is also coupled with an understanding of the organisation of care within a range of primary care practices and policies. Existing research data suggests that the organisation of care for patients with frequent attendance is an important ingredient to intervention success [36,37,70]. The current emphasis in terms of policy and financial incentives on individual procedures and activity as opposed to delivery of integrated care within practices and across organisations [73] may be problematic to tackling frequent attendance in primary care [50].

Thus a complex intervention delivered in English primary care is likely to involve changes in the organisation of care for these patients, incentives to manage them, training of primary care professionals and support from mental health professionals. The acceptability, feasibility and clinical utility of cognitive behaviour formulation and treatment for patients with persistent frequent attendance will be explored. These data will only provide information on one aspect of the intervention and information to plan a further evaluation of the treatment approach. However, the definition of the economic and clinical need for an intervention, theoretical development and testing of both cognitive behaviour therapy and organisational care issues, and early piloting of an intervention element (cognitive behaviour therapy formulation and treatment) will together provide much needed data for development of a more complex multifaceted intervention involving training of GPs and organisation of practice care [57].

## Abbreviations

BMI: Body mass index; CBT: Cognitive behaviour therapy; CSRI: Client Service Receipt Inventory; EQ5D: Euroqol quality of life five dimension; FA: Frequent attender; GAD-7: Generalised Anxiety Disorder Assessment, 7 item; GLM: Generalised linear model; GP: General practitioner; HAI: Health Anxiety Inventory; INR: International normalised ratio; MUS: Medically unexplained symptoms; NHS: National Health Service; PBR: Payment by results; PHQ-9: Patient Health Questionnaire, 9 item; PHQ-15: Patient Health Questionnaire, 15 item; SCID-1: Structured Clinical Interview for Diagnostic and Statistical Manual of Mental Disorder, Fourth Edition, Axis 1 Disorder; SF-36: Short, Form Medical Survey, 36 item.

## **Competing interests**

The authors declare that they have no competing interests.

## **Authors’ contributions**

All authors contributed the design of the study, the study protocol and the writing up of the paper. All authors read and approved the final manuscript. RM leads the project and wrote the project application for funding with JK and AA. MS and TG manage the project on a day to day basis. SP and MS collect the day to day data. MF leads the resource use from medical records. health economics data extraction. MJ leads the health economics analysis and MY leads the statistical analysis. CA and SM perform the cognitive behaviour therapy and with RM the CBT formulation analysis. The qualitative analyses are carried out by SB, MS, JK, RM and RMcD.

## Pre-publication history

The pre-publication history for this paper can be accessed here:

http://www.biomedcentral.com/1471-2296/13/39/prepub
